# Biomarkers of the Metabolic Syndrome and Breast Cancer Prognosis

**DOI:** 10.3390/cancers2020721

**Published:** 2010-04-28

**Authors:** Qiu-Li Zhu, Wang-Hong Xu, Meng-Hua Tao

**Affiliations:** 1 Department of Epidemiology, School of Public Health, Fudan University, Shanghai 200032, China; 2 Department of Social and Preventive Medicine, School of Public Health and Health Professions, University at Buffalo, Buffalo, NY 14214, USA

**Keywords:** metabolic syndrome, breast cancer, epidemiology, biomarkers, prognosis

## Abstract

In spite of its public health importance, our understanding of the mechanisms of breast carcinogenesis and progress is still evolving. The metabolic syndrome (MS) is a constellation of biochemical abnormalities including visceral adiposity, hyperglycemia, hyperinsulinemia, dyslipidemia and high blood pressure. The components of the MS have all been related to late-stage disease and even to a poor prognosis of breast cancer through multiple interacting mechanisms. In this review, we aim to present a summary of recent advances in the understanding of the contribution of the MS to breast cancer with the emphasis on the role of biomarkers of the MS in the prognosis of breast cancer.

## 1. Introduction

Breast cancer is the most common cancer affecting women. Although many risk and prognostic factors of breast cancer have been established, and numerous biomarkers have been linked to breast cancer, our understanding of breast cancer prognosis is still evolving. In recent years, evidence has rapidly accumulated on the potential role of multiple metabolic disorders in the development and progress of breast cancer. The metabolic syndrome (MS), a cluster of metabolic disorders that are the known risk factor of cardiovascular disease and diabetes, has been proposed to play a critical role in the risk [1] and prognosis of breast cancer [2].

There are several commonly used definitions of the MS [3,4,5], including a newly-developed harmonized one [6]. These definitions, although with slightly different emphasis, can be distilled into the presence of at least three metabolic abnormalities among central obesity, dyslipidemia (high triglycerides or low HDL-cholesterol levels), hyperglycemia and elevated blood pressure. The four features have been demonstrated to be closely related to breast cancer risk [7,8,9,10,11,12], and some have been identified to be associated with late-stage of the disease and a poor prognosis [13,14,15,16]. Meanwhile, the effect of these metabolic disorders on breast cancer survival can be modified by multiple factors [16,17,18,19]. As the MS has experienced an abrupt increase in recent decades [20], simultaneously the number of female breast cancer survivors continues to rise globally; identifying the modifiable biomarkers of the MS and breast cancer and the possible mechanism is of particular interest. In this review, we report on current understanding of the contributions of the MS to the prognosis of breast cancer with emphasis on the role of the biomarkers of the four most important features. We also aim to present a summary of possible underlying mechanisms and the possible approaches to improve the breast cancer prognosis through controlling the MS.

## 2. Obesity and Breast Cancer Survival

Generally, obesity, estimated by body weight, body mass index (BMI), or waist-to-hip ratio (WHR), is positively associated with breast cancer risk in postmenopausal women, while inversely related to the risk in premenopausal women. The effect of obesity on the prognosis of both pre- and post-menopausal breast cancer has aroused increasing interest in recent years, and has immense public health implications.

Body weight is a direct biomarker of obesity. Previous studies have found that heavier women diagnosed with breast cancer are more likely to experience poorer survival and have an increased likelihood of recurrence of the disease. Donegan *et al.* [13] first reported that, among 2,627 breast cancer cases, recurrence rates were much higher among breast cancer patients who weighed more than 130 pounds compared to the leaner cases. Similar adverse effects of increased body weight on the survival of breast cancer have been reported in the majority of later studies over the past three decades [21,22,23]. A meta-analysis found that for increased body weight, the hazard ratios (HR) were 1.78 (95% CI, 1.50–2.11) and 1.36 (95% CI, 1.19–1.55) for recurrence risk after five years of breast cancer diagnosis and death of the disease at 10 years, respectively [24]. The negative effects of body weight on breast cancer prognosis were observed in both pre- and post-menopausal women [21,22,23,25,26], and some studies even reported stronger associations in premenopausal women [25,26]. However, some of earlier studies did not find any association of body weight with subsequent recurrence and/or survival [27,28]. These reported differences may be due to the relative small sample sizes of some studies or alternatively if menopausal status and other confounding factors such as body fat distribution were not taken into consideration [21,23,27]. Furthermore, most previous studies only focused on the association of body weight measured at the time of diagnosis with breast cancer prognosis, and overlooked the effects of weight changes after diagnosis and during the treatment on recurrence and survival [26].

BMI, measured as weight (kg)/height (m^2^), is another biomarker of obesity, and increasing BMI is related to prognosis of breast cancer as shown in extensive reviews in recent years [22,29,30,31,32,33]. In an early study by Greenberg *et al.* [25], BMI was not related to premenopausal breast cancer survival. However, in a population-based follow-up study of 1,177 young breast cancer patients (<45 years), women in the highest quartile of BMI were 2.5 times more likely to die from the disease within five years of diagnosis compared with women in the lowest quartile [34]. It was also found that these heavier women tended to have larger tumor size, higher histological grade, and were more likely to have markers of high cellular proliferation than the thinner women [34]. Other studies reported similar associations between BMI at the time of diagnosis and poor outcomes among premenopausal women with breast cancer [26,35,36]. Most, but not all, studies have confirmed the association between BMI and breast cancer recurrence and survival in postmenopausal women [28,31,33,37]. Different from the previous studies, a recent large-scale cohort of older breast cancer survivors (≥65 years), the Study of Osteoporotic Fractures, showed an age-dependent relationship between BMI and survival among postmenopausal breast cancer [38]. At age 65 and 70 years, women with higher BMI had an increased risk of breast cancer mortality compared with women with a BMI of 22.6; whereas, there was a reverse association between BMI and the outcome among women aged 80 and 85 years.With the growth of aging population in the worldwide, management of breast cancer among the elderly has been a significant public health problem, and further studies on elderly breast cancer survivors are needed.

Increasing BMI has also been associated with a poorer prognosis among women with early stageand invasive primary breast cancer [39,40,41]. The breast cancer patients with no positive nodes and being in the highest quartile of BMI (>29) had an increased risk of death from the disease than those in the lowest quartile (HR = 2.5, 95% CI, 1.2–5.2) [39]. Similar associations between obesity and poor breast cancer prognosis also have been reported in Asian or African American populations [15,42,43,44,45]. Compared with white breast cancer patients, African-American patients are more likely to have a worse prognosis, which may be at least partially related to the higher prevalence of obesity in African-Americans [43,45]. The relationship between overweight/obesity and breast cancer survival and recurrence has also been demonstrated in Asian populations, which have the lowest breast cancer mortality rates internationally [15,42,44]. Tao *et al.* [15] found that BMI was associated with increased risks of death, and the effect of obesity was stronger among post- than pre-menopausal Chinese women. Recently, another cohort study in China reported that breast cancer patients with a BMI ≥ 30 at diagnosis had the HRs of total mortality of 1.55 (95% CI: 1.10–2.17) and relapse/disease-specific mortality of 1.44 (95% CI: 1.02–2.03) compared with patients with normal BMI [42].

Weight gain after diagnosis, especially among breast cancer patients with systematic adjuvant therapy (*i.e.*, chemotherapy and tamoxifen use), has been frequently reported [22,46,47]. In a cohort of 5,014 women with early-stage breast cancer, approximately 26%, 37% and 33% of breast cancer survivors gained ≥5% of their at-diagnosis body weight during the first 6, 18 and 36 months after diagnosis, respectively, and more weight gain was observed among those who had a more advanced disease stage, were younger, had lower BMI at diagnosis, were premenopausal, or received chemotherapy or radiotherapy during the first six months after cancer diagnosis [46]. In recent years, some studies have investigated the effects of weight gain after breast cancer diagnosis on survival and mortality in different populations [22,30]. It was reported that each 5-kg weight gain after breast cancer diagnosis was associated with a 13% increase in breast cancer-specific mortality [48]. In another study by Camoriano *et al.* [63], premenopausal breast cancer patients who gained more than 5.9 kg were 1.6times more likely to die from the disease than women who gained less. Chen *et al.* [42] recently reported similar findings. Furthermore, results from the Nurses’ Health Study showed that the association of weight gain after breast cancer diagnosis and increased breast cancer mortality was limited among women who were of normal weight (BMI < 25) before diagnosis [26]. Results from these studies suggest that efforts to maintain or decrease body weight after a breast cancer diagnosis is very important for breast cancer survivors, even for those who were overweight before diagnosis; breast cancer patients, particularly those receiving adjuvant chemotherapy, may benefit from maintaining or decreasing weight after breast cancer diagnosis through balancing energy intake (diet) and consumption (physical activity) [49,50].

As a commonly used anthropometric indicator for abdominal obesity, WHR has been evaluated as a critical biomarker for breast cancer survival by a number of studies in recent years [14,15,36,42,51]. In a study of 603 breast cancer patients (357 postmenopausal), Borugian *et al.* [14] reported a strong positive association between WHR and breast cancer mortality only in postmenopausal women. Results from another large-scale cohort study showed that the highest quartile of WHR was associated with increased mortality among young breast cancer cases aged 20–54 [36]. Similar association between high WHR and poor breast cancer survival was confirmed in a follow-up study [51]. However, the Iowa Women’s Health Study did not find any relationship between WHR before diagnosis and survival of postmenopausal women with breast cancer [52]. Results from two follow-up studies of primary breast cancer patients in China showed no significant relationship between WHR or waist circumference and breast cancer survival and mortality in Chinese women either [15,42]. The differences between these observations may be partly due to the ethnic discrepancy in the body composition profile across different populations. More studies are needed to evaluate the influence of the abdominal obesity on the prognosis of breast cancer, while considering the potential impacts of the use of hormone replacement therapy, breast tumor characteristics, and treatment after the disease diagnosis.

Several possible mechanisms have been hypothesized to account for the poorer prognosis of breast cancer in obese women. Obesity usually makes the tumor harder to be detected at an early stage. Consequently, obese women tend to be diagnosed at a more advanced stage, and thus have an increased likelihood of treatments failing [53]. Secondly, the higher endogenous levels of estrogen, insulin and triglycerides in obese women may accelerate the growth and metastasis of the tumor. In overweight/obese women, there may be enhanced conversion in the adipose tissue of the estrogen precursor, androstenedione, to estrone, which can accelerate the tumor growth [54,55]. After menopause, the adipose tissue predominately produces estrogen with concomitant increasing concentration of triacylglycerol and insulin, which may result in prolonged exposure to increased and more biologically active forms of estrogen in overweight postmenopausal women [56]. Compared with women with low WHR, women with high WHR have lower serum sex hormone binding globulin (SHBG), higher free testosterone, and possibly higher estrogen levels [55,57]. The observed abdominal obesity-survival association may be due to elevated concentrations of estrogen, as well as high levels of insulin and triglycerides [58]. Some studies have suggested that obesity is a marker for insulin resistance and hyperinsulinemia [59,60], whose role in breast cancer survival will be discussed in the next section. In addition, obesity is an index of positive energy balance characterized with excess fat intake or lack of physical activity, which may act as an adverse contributor to poor prognosis of breast cancer [50,52]. Furthermore, obesity, particularly central obesity, could induce chronic low-grade inflammation [61], which is another known risk factor of breast cancer and can increase the likelihood of epigenetic alterations such as aberrant DNA methylation [62,63]. Aberrant DNA methylation plays a crucial role in breast carcinogenesis [64,65], and shows promise as a potential biomarker in breast cancer early detection and prognosis [66,67]. Breast cancer is heterogeneous; a better understanding of the mechanisms and influences of epigenetic changes may lead to better treatment and improved survival of certain subtypes of breast tumor.

Although the underlying mechanisms have not been completely understood [14,52], obesity is a known factor of the poor prognosis of breast cancer. Because obesity can be modifiable through proper diet and physical activity throughout the lifetime, weight management provides an important opportunity to decrease mortality and improve quality of life for women with breast cancer.

## 3. Hyperinsulinaemia, Hyperglycemia and Type 2 Diabetes Mellitus with Breast Cancer Outcome

Insulin resistance, a central characteristic of the MS defined by WHO, is a state in which some organs become resistant to the effect of insulin that is needed to shuttle glucose into cells. To compensate for the resistance to insulin, the pancreas produces more insulin, which leads to an increase in circulating levels of insulin. The compensation may continue for many years, but the pancreas cannot maintain this high insulin output indefinitely, especially in some susceptible individuals. The compensatory hyperinsulinaemia and the subsequent hyperglycemia due to insulin resistance are believed to be the origin of the MS and type 2 diabetes mellitus and a crucial contributor to breast cancer prognosis.

Hyperinsulinemia and hyperglycemia are biomarkers for insulin resistance [68]. Both of these disorders are critical to the initial development and progression of breast cancer. Berrino *et al.* [69] found that, after adjusting for hormone receptor status and tumor stage at diagnosis, serum glucose was significantly higher in patients who had a recurrence than those who did not in a prospective study. More evidence is available for hyperinsulinemia. Goodwin *et al.* [70] firstly reported that in both premenopausal and postmenopausal women, insulin levels were correlated with breast tumor stage, nodal stage and tumor grade, and related to an increased risk of distance recurrence and a shorter survival regardless of the BMI. Bozcuk *et al.* [71] found that the fasting serum insulin level was an independent predictor for overall survival in metastatic breast cancer patients. Similar findings were subsequently reported by Pasanisi *et al.* [2] and Pollak *et al.* [72], both of them observed a positive association of high levels of insulin or C-peptide, a subunit of insulin, with breast cancer mortality.

Although whether hyperinsulinaemia and hyperglycemia increase the risk of breast cancer recurrence and breast cancer specific mortality is not clear, multiple mechanisms through which the conditions elicit adverse effects have been proposed. High circulating levels of glucose contribute to the poor prognosis of breast cancer, possibly by providing abundant energy for proliferation of a neoplastic cell, cultivating an amiable environment for the growth of malignant cell clones and fostering cancer development [73]. Moreover, concentration of glucose is mainly regulated by insulin, a growth factor that can stimulate the growth of the tumors directly and indirectly [74]. Insulin has an important mitogenic effect and can signal growth directly through, at least in part, its own receptors [75,76]. In cell culture, insulin induces a dose-dependent growth response in breast cancer cell lines acting via the insulin receptor [77,78], which has been demonstrated to be almost ubiquitously present in human breast cancer and to have prognostic significance [79]. The insulin receptor has been related to tumor size [80], grade [80], and mortality of breast cancer [81]. Furthermore, insulin is highly regulated by endogenous sex hormones [82], particularly by estrogens - the hormone involved in the promotion and growth of breast cancer [83]. Hyperinsulinemia has generally been related to an inhibition of aromatase activity [84], suppressed SHBG levels [85] and thus elevated both free and combined available estrogen concentrations. More importantly, insulin can interact and synergize with other growth promoting changes such as the insulin growth factor (IGF) signaling system.

Hyperinsulinaemia could specifically augment IGF-1 levels and make cells more sensitive to the growth factor. IGF-I is a small peptide (7,500 Da) with significant structural homology with proinsulin and insulin [86]. It is the main growth factor that inhibits apoptosis and stimulates cell proliferation after puberty [87]. IGF-1 has the nature to stimulate multiple cellular responses that are related to growth such as synthesis of DNA, RNA, and cellular proteins [88] and induceing metastasis in many types of malignancies [89]. The IGF signaling system also interplays with estrogen activity on many levels in the development and progression of breast cancer [90,91,92]. Moreover, it is possible that the IGF system elicits adverse effects in the prognosis of breast cancer by inducing anti-cancer drug resistance [93] and up-regulating expression of several genes that are involved in transport and biosynthesis of amino acids [94]. High circulating levels of IGF-I has been linked to poorer prognosis of breast cancer [95,96,97], although the evidence is inconsistent [98,99,100]. IGF receptor I expression in primary breast cancer has also been suggested as an independent favorable prognostic factor, while IGF binding protein-3 (IGFBP-3) expression is associated with a poor outcome of breast cancer [101]. Recently, IGFBP-2 has been shown as another independent and positive predictor of overall survival of breast cancer [92].

Insulin resistance and hyperinsulinaemia are also involved in prognosis of breast cancer by inducing several other changes, such as increased inflammation [102] and elevated adipocytokines, which have been related to angiogenesis [103]. Therefore, hyperinsulinaemia may be most beneficially viewed as one strand in a network of interacting disturbances that promote the development and progression of cancer.

In recent years, type 2 diabetes mellitus, a complex disease characterized by hyperglycemia, hyperinsulinemia, insulin resistance, obesity and other metabolic abnormalities, has been related to breast cancer prognosis. Diabetic patients have experienced higher mortality and recurrence rates after diagnosis and treatment for breast cancer. By analyzing the data from the Surveillance Epidemiology and End Results (SEER) cancer registry, Yancik *et al.* [104] found that breast cancer patients with diabetes were more likely to die prematurely from breast cancer than were patients without diabetes (RR = 1.76; 95% CI: 1.23, 2.52). Verlato *et al.* [105] observed a higher risk of death from breast cancer in diabetic women than among the general population (HR = 1.40; 95% CI: 1.06, 1.81) in a cohort of 3,782 diabetic women in northern Italy. Wolf and colleagues [106] reported that diabetic patients present with breast cancer had adverse characteristics such as more advanced stage, larger tumor and negative status of hormone receptors. The association could not be attributed to parity, family history of breast cancer, and was independent of obesity, indicating that diabetes may have an independent effect on cancer prognosis. A meta-analysis of five cohort studies on diabetes and mortality from breast cancer yielded a summary RR of 1.24 (95% CI, 0.95–1.62) for women with diabetes *versus* those without, although only three out of five observed a significant association [107]. In the largest study with 588,321 subjects, RR of breast cancer mortality for diabetic women was 1.27 (95% CI, 1.11–1.45) compared with the non-diabetic females after adjusting for age, race, BMI, physical activity, smoking, and alcohol [108]. A more recent retrospective cohort study linked diabetes with a close to 40% increase in mortality within the first five-year following breast cancer [16]. In this study, however, the cause of death was not recorded and diabetic women without breast cancer also had an increase in mortality, suggesting that diabetes rather than breast cancer was the major contributor to the increase in mortality. Another meta-analysis also observed an increased mortality HR of 1.61(95% CI, 1.46–1.78) for breast cancer with pre-existing diabetes mellitus [109]. More recently, Patterson *et al.* [110] observed over two-fold increased risk of additional breast cancer mortality in participants with a history of early stage breast cancer and diabetes (HR = 2.5, 95% CI: 1.4, 4.4). Tseng *et al.* [111] observed a 37–43% increase in breast cancer mortality in diabetic women in all age groups by comparing the secular trend for breast cancer mortality rates in the general population and in diabetic women in Taiwan.

Interestingly, evidence from an intensive care study indicates that achieving glucose control may lead to better clinical outcomes of breast cancer [112]. An animal study showed that insulin sensitizing treatment is sufficient to abrogate type 2 diabetes-mediated mammary tumor progression [113]. The finding implicates a promising role of early administration of insulin-sensitizing therapy in prolonging survival of breast cancer patients with type 2 diabetes mellitus. Goodwin and colleagues [114] have administered Metformin, an oral anti-diabetic drug, to lower insulin levels in women with early breast cancer, and are trying to evaluate the effect of the novel approach on breast cancer outcomes in the later stage of the clinical trial.

As mentioned above, several mechanisms have been put forth for the adverse effect of hyperinsulinaemia in the progression of breast cancer. However, it remains unclear whether diabetes can make the cancer grow more aggressively or promote the sensitivity of the host organism to cancer progression through these mechanisms. Currently, the comorbidity and interaction of diabetes with breast cancer is arousing great research interest. It is supposed that the presence of diabetes may affect the therapy of the breast cancer. While anti-diabetic drugs have a minor influence on cancer risk [115], drugs used to treat cancer may either worsen pre-existing diabetes [116] or increase chemotherapy-related toxicities [117]. Therefore, it is possible that diabetic patients have to receive lower chemotherapy doses because the clinicians may consider the cardiac, renal, and neurologic complications commonly associated with diabetes when they treat breast cancer. Ultimately, the outcome for cancers may be worsened by the avoidance of agents that have been shown to provide the best clinical response and survival in cancer patients without these disease complications. It has been shown that diabetic cancer patients were frequently treated less aggressively and had a worse prognosis compared to those without diabetes in a large population based analysis [118]. It is also possible that diabetic patients may have a worse response to chemotherapy compared with non-diabetic individuals [93].

In conclusion, chronic hyperinsulinemia, either with or without clinically manifest type 2 diabetes mellitus, is a possible factor favoring cancer progression due to the mitogenic effect of insulin. It needs to be stressed, however, that no published studies have related type 2 diabetes mellitus, hyperinsulinemia, or insulin resistance specifically to breast cancer outcome. The complex and multifactor-driven role of hyperinsulinemia in breast cancer prognosis has warranted further studies including clinical trials to understand the nature of their relationship, particularly as the general population ages and the magnitude of both health problems continues to grow.

## 4. Dyslipidaemia and Prognosis of Breast Cancer

Dyslipidaemia refers to an elevation in the concentrations of total cholesterol, the low-density lipoprotein cholesterol (LDL-C) and the triglyceride (TG) concentrations, and a reduction in the high-density lipoprotein cholesterol (HDL-C) in the blood. It often coexists with high levels of serum insulin and obesity [119]. As two important components of the MS [120], higher TG and lower HDL-C levels in serum were found to be more common in patients with malignant diseases including breast cancer compared with non-cancer subjects [121,122]. Some earlier studies have reported the prognostic effect of serum cholesterol on the survival of breast cancer [21]. Later, results from a study by Vatten *et al.* [123] showed that breast cancer patients in the highest quartile of the preclinical total serum cholesterol had an increased risk of dying from the disease compared to women in the lowest quartile (HR = 2.0, 95% CI, 1.1–3.). A large scale prospective study, however, did not find significant correlation between serum cholesterol level and breast cancer survival among both younger (aged < 50 years) and older (aged ≥ 60 years) patients [124]. In a recent cohort study of 520 early-stage breast cancer patients, after adjusting for age, tumor-related variables and BMI, a trend towards increased risk of recurrence with higher total cholesterol was observed, although no significant associations between fasting TG and breast cancer recurrence or death was found [125]. These findings suggest that the different fractions of cholesterol may contribute different influences to the relation between dyslipidaemia and breast cancer prognosis.

Previous studies found that the turnover of TG was faster in breast cancer tissue than in the adjacent normal tissue, indicating a significant difference in TG metabolism between the mammary tissues [126,127]. Some studies also reported that women with relative androgen excess (such as polycystic ovary syndrome) have lower levels of serum HDL-C, a suggested marker of androgen status [128], compared with those having normal ovarian function [129]. Low HDL-C is further related to increased levels of several other hormones including estrogens, insulin, and IGF-I, all of which can stimulate cancer development [130]. The positive association between low HDL-C and breast cancer risk may reflect the relative importance and mutual dependence of different pathways in the progression of breast cancer, particularly among postmenopausal women. For postmenopausal women, bio-available estrogens, the major stimulus for breast carcinogenesis, are mainly formed in fat tissue or in the granulosa cells of the ovarian follicle through the aromatization of androstenedione and testosterone instead of direct ovarian estrogen production [131]. On the other hand, higher TG and lower HDL-C levels have been constantly found to be correlated with insulin resistance and type 2 diabetes mellitus [132,133], and thus adversely affect the prognosis of breast cancer. Despite these possible explanations, the mechanisms by which dyslipidemia affects survival of breast cancer are still not well known, and further studies are needed.

## 5. Hypertension and Prognosis of Breast Cancer

While a large amount of studies have evaluated the effects of obesity, hyperglycemia and dyslipidemia on the prognosis of breast cancer, evidence for the influence of hypertension is still very limited. So far, the association between hypertension and breast cancer survival has been investigated in a few studies with inconsistent results. Results from a prospective study by a 19-year follow-up of 11,075 women showed that women who had hypertension at baseline had slightly increased total mortality from cancer (HR = 1.10, 95% CI, 0.93–1.31); however, no association with breast cancer mortality was observed [134]. A recent study found that the prevalence of hypertension was much higher in African-American breast cancer patients (63.4%) than that in white patients (35.5%), and the presence of hypertension before breast cancer diagnosis was associated with worse survival, particularly in African-American women [135]. Recently, Braithwite *et al.* [136] evaluated the effect of hypertension as an important comorbidity on breast cancer survival in 416 African-American and 838 white women. The presence of hypertension before breast cancer diagnosis was independently related to all cause survival with the HR of 1.33 (95% CI, 1.07–1.67), and it accounted for 30% survival disparity between African-American and white women diagnosed with breast cancer. Results from the above two studies suggested that control of hypertension comorbidity may help to improve the overall survival of African-American breast cancer patients and reduce racial disparity.

Results from both animal models [137,138] and human studies [139] have implicated that hypertension may increase the response to carcinogens and initiate the process of carcinogenesis. The potential mechanisms for the adverse impact of hypertension on the survival of breast cancer, however, are much less clear. Insulin resistance may explain part of the possible association, because insulin and/or insulin resistance are hypothesized to be associated with hypertension [140] by contributing to the pathogenesis of the disorder [141]. However, the evidence was also controversial, with a strong [140,142] and null association between hypertension and insulin [143]. More evidence is needed to elucidate and to clearly understand the association between hypertension and breast cancer survival.

## 6. Summary

As described previously, the biomarkers for each individual component of the MS have been indicated to be associated with breast cancer survival. It is plausible that the MS, a cluster of these metabolic disorders, is associated with important clinical features of breast cancer and may act as a predictor for breast cancer prognosis. Recently, Healy *et al.* [102] reported that the MS was associated with more aggressive postmenopausal breast tumor biology. Patients with a later pathological stage (II-IV) were significantly more likely to be obese, centrally obese, hyperglycaemic and hyperinsulinaemic. As a result, the prevalence of the MS was higher (51%) in advanced stage disease than in early stage disease (12%). Patients with node-positive disease were also significantly more likely to be hyperinsulaemic and have the MS than patients with node-negative disease. Till now, however, there are still few studies to examine the relationship of breast cancer survivorship with the MS as a group of abnormal symptoms. After follow-up 110 postmenopausal breast cancer patients for 5.5 years, Pasanisi *et al.* [2] found that the HR of subsequent recurrence of breast cancer was 3.0 (95% CI, 1.2–7.1) for those diagnosed with the MS at baseline.

In conclusion, the MS may play an important role in the prognosis of breast cancer mediated by insulin resistance, a state that is highly regulated by sex-hormone pathway and can stimulate growth of malignant cells directly and indirectly through IGF signal pathway [144]. Since both breast cancer and the MS are of polygenic and multi-factorial origin and usually in comorbidity, their relationship is definitely complex. If the role of the biomarkers of the MS in breast cancer survival is confirmed, it may have an important implication in predicting and improving survival of breast cancer. The MS and individual metabolic disorder can be prevented and modified by adopting healthy lifestyles; therefore, it is possible to improve breast cancer prognosis through taking balanced diet, increasing physical activities, controlling body weight [145,146,147], and potentially by administrating early insulin reducing therapy [145,146,147]. There is a compelling need to carry out more long-term prospective studies and large scale intervention trials with better design to evaluate both the short- and long-term effects of the MS on breast cancer outcomes, to elucidate the preventive value of changes in lifestyle, and to better understand the potential mechanisms.
